# Disparities in receipt of recommended care among younger versus older medicare beneficiaries: a cohort study

**DOI:** 10.1186/s12913-017-2168-5

**Published:** 2017-03-29

**Authors:** Ling Na, Joel E. Streim, Liliana E. Pezzin, Jibby E. Kurichi, Dawei Xie, Hillary R. Bogner, Pui L. Kwong, Steven M. Asch, Sean Hennessy

**Affiliations:** 1 Center for Clinical Epidemiology and Biostatistics, Perelman School of Medicine, University of Pennsylvania, Philadelphia, PA USA; 2 Geriatric Psychiatry Section of the Department of Psychiatry, Perelman School of Medicine, University of Pennsylvania, Philadelphia, PA USA; 3 Center for Patient Care and Outcomes Research (PCOR), Medical College of Wisconsin, Milwaukee, WI USA; 4 Department of Medicine, Medical College of Wisconsin, Milwaukee, WI USA; 5 Division of General Medical Disciplines, Stanford University School of Medicine, Menlo Park, CA USA; 6 Center for Pharmacoepidemiology Research and Training, University of Pennsylvania, Philadelphia, PA USA

**Keywords:** Medicare, Younger beneficiaries, Health disparity, Recommended care, Quality of care

## Abstract

**Background:**

Although health disparities have been documented between Medicare beneficiaries based on age (<65 years vs. older age groups), underuse of recommended medical care in younger beneficiaries has not been thoroughly investigated. In this study, we aim to identify and characterize vulnerabilities of the younger Medicare age group (aged <65 years) in relation to older age groups (aged 65–74 years and ≥75 years) and to explore age group as a determinant of use of recommended care among Medicare beneficiaries.

**Methods:**

We conducted a cohort study of community-dwelling Medicare beneficiaries who participated in the Medicare Current Beneficiary Survey between 2001 and 2008 (*N* = 30,117). Age group characteristics were compared using cross-sectional data at baseline. During follow-up, we assessed the association between age and receipt of recommended care on 38 recommended care indicators, adjusting for sociodemographic and clinical characteristics. Follow-up periods differed by component indicator.

**Results:**

At baseline, a higher proportion of younger beneficiaries experienced social disadvantage, disability and certain morbidities than older age groups. During follow-up, younger beneficiaries were significantly less likely to receive overall recommended care compared to those 65–74 years of age (adjusted odds ratio and 95% confidence interval: 0.75, 0.70–0.80). In addition, male gender, non-Hispanic black race, less than high school education, living alone, with children or with others, psychiatric disorders and higher activity limitation stages were all associated with underuse of recommended care.

**Conclusions:**

Younger Medicare beneficiary status appears to be an independent risk factor for underuse of appropriate care. Support to ameliorate disparities in different social and health aspects may be warranted.

## Background

The Healthy People 2020 initiative seeks to eliminate health disparities and improve the health of all groups in the US [1]. A distinct group that suffers multiple health disparities, yet has not been investigated thoroughly, is Medicare beneficiaries under 65 years of age. Younger Medicare beneficiaries face major social disadvantages and a disproportionately high burden of disabilities and medical morbidities. Unlike those who are eligible for Medicare solely due to being 65 years of age and older, younger enrollees must have received Social Security disability benefits for 24 months or have either amyotrophic lateral sclerosis or end-stage renal disease [2]. Younger Medicare beneficiaries are more likely to be male, non-white, economically and educationally disadvantaged, to be in fair or poor health, and to have a higher prevalence of disabilities and mental health disorders [3–6]. In 2012, younger beneficiaries constituted 17% of the 50.8 million Medicare enrollees, but triggered 20% of total Medicare expenditures [7]. Despite these higher expenditures, they underutilized preventive health services including influenza vaccine, eye and dental exams, mammograms, and prostate exams [4].

Braveman’s health disparity framework lays the ground for our analysis of younger Medicare beneficiaries [8]. A health disparity is a population-specific, potentially avoidable difference in health or important influences on health that is systematically associated with socially disadvantaged groups [8], such as the impoverished, racial minorities and individuals with disabilities. An important way to eliminate health disparities is through equitable health care, defined as equally accessible care to all users, and greater provision of care to users who demonstrate greater need [8–10]. In Braveman’s framework, a health disparity should be assessed by comparing groups in a social hierarchy in relation to each other [11], because such comparisons help policy makers identify vulnerable social groups, target interventions and reallocate resources to achieve greater health equity. Factors associated with health disparities include minority race [12, 13], lower income and less education [14], and disability [15–17]. Often these vulnerabilities, as well as rural location and reduced physician supply, are also associated with poor quality of care [18–25]. Although it is expected that younger beneficiary status is associated with health disparity due to Medicare enrollment criteria, younger beneficiaries demonstrated largely unmet health care needs.

However, younger beneficiaries are often excluded from studies of Medicare beneficiaries. The few pioneering studies comparing younger versus older beneficiaries highlighted the importance of the topic, although they tend to have several limitations [4–6]: self-reported health service utilization is subject to recall bias; types of services are often limited to preventive care; and crude associations without risk adjustment are not particularly useful for policy planning. To better capture underuse of care in the younger population, we employed claims data, a variety of indicators and risk-adjusted models. Furthermore, three main characteristics of younger beneficiaries (greater comorbidity, disability and socioeconomic disadvantages) do not always affect quality of care in the same direction. Multimorbid patients tend to get higher quality of care [26], disability has mixed quality [24]; minority race and lower income, while also having mixed quality, tend to predict worse care [21, 27]. Comparing younger with older Medicare beneficiaries can shed light on the direction and magnitude of these relationships, and their synergies and dys-synergies as they co-occur in younger beneficiary population. The comparison is important as a policy evaluation issue: is the Medicare program failing its younger beneficiaries?

We sought to identify predictors of underuse of recommended care by applying Asch’s underuse indicator system to recent Medicare claims of health service utilization [21]. Asch’s underuse indicator system is a clinically valid, comprehensive and claim-based measurement tool, which examines highly prevalent conditions and preventive care. These indicators have been validated on both inpatient, outpatient and physician service claims data for Medicare beneficiaries 65 years of age and older [21, 24], but not younger beneficiaries. Therefore, we aimed to characterize vulnerabilities of the younger Medicare age group and then explore age group as a determinant of use of recommended care among Medicare enrollees. We assess the extent to which the earlier findings of disparities in sociodemographic and health characteristics hold in younger beneficiaries in our data. We further test our hypothesis that compared to older beneficiaries, younger beneficiaries are less likely to receive recommended care after accounting for sociodemographic characteristics, degree of comorbidity and activity limitation.

## Methods

### Study sample

We analyzed data from a nationally representative sample of the Medicare population, the Medicare Current Beneficiary Survey (MCBS) [28, 29]. The MCBS is a longitudinal panel survey that contains individual-level information of sociodemographics, health care encounters and health and physical functioning. Survey participants are typically interviewed three times per year for 4 years with health and functioning assessed in the fall of each year. The sample is replenished annually with newly enrolled beneficiaries replacing those who died or exited the survey. Survey data are linked to Medicare claims data that are available for 3 years after the initial survey. The MCBS uses multistage sampling design, with weights, strata and cluster information available. MCBS oversamples beneficiaries aged 85 years and older and those aged 65 years and younger. One study reported that the initial response rate of MCBS was 82.6%, similar to other national surveys [29]. The response rates were 82–83% across different age categories. The magnitude of potential bias due to non-response was reduced by non-response adjustment provided in the survey [29]. Our study included community-dwelling Medicare beneficiaries who enrolled in the MCBS between 2001 and 2008. The entry panels of 2001–2007 were followed for 3 years, and panel 2008 was followed for 2 years because claims data beyond 2010 were not available.

The study was approved by the University of Pennsylvania Institutional Review Board.

### Receipt of recommended care

To assess receipt of recommended care, we adapted the indicator system measuring underuse of necessary care that was developed by Asch and colleagues [21] and later modified by Chan [24]. The original indicators span several domains of care: initial evaluation, diagnostic tests, therapeutic interventions, hospitalization follow-up, monitoring of routine care and avoidable outcomes. The indicator system was tested and validated on 1992–1993 Medicare claims and was applied to 1994–1996 claims data [21]. After excluding six avoidable outcome indicators (because we wished to focus on process measures) and three indicators with inadequate sample size, we adapted 38 indicators or process measures, of recommended care to our study. Three of these 38 indicators measured receipt of preventive care: a physician annual visit, a biennial visual impairment assessment, and a biennial mammography for women aged between 45 and 75 years. The remaining 35 indicators examined care for acute and chronic conditions, including acute myocardial infarction, anemia, angina, breast cancer, cerebrovascular accident (CVA), transient ischemic attack (TIA), cholelithiasis, chronic obstructive pulmonary disease (COPD), congestive heart failure (CHF), depression, diabetes, gastrointestinal bleeding and hypertension.

Each indicator specified which beneficiaries were eligible (i.e., had an opportunity) for its assessment, the care that should be received, and a recommended time interval. Receipt of recommended care was coded as present if claims data indicated delivery of care within the recommended time frame, and absent otherwise. Receipt of care was assessed at the opportunity level; thus a beneficiary might have multiple opportunities for recommended care. Opportunities were not eligible for indicator assessment if they had incomplete follow-up time due to death or loss to follow-up, disenrollment in Part A and/or part B, or enrollment in a managed care program during the assessment period. For indicators with short assessment periods (2–4 weeks), subjects were excluded if there was a hospitalization or ER visit during the follow-up period.

### Age groups

Our main interest was Medicare beneficiaries younger than age 65. Recognizing the potential heterogeneity of older beneficiaries in their health status and health care quality [5], we classified them as younger old (65–74 years) and older old (75 years and older).

### Sociodemographic and clinical characteristics

Sociodemographics and clinical characteristics were assessed based on self- or proxy-report in the surveys. Sociodemographics included sex, race (non-Hispanic white, non-Hispanic black, Hispanic or other), education (less than high school education or high school diploma and above), dual enrollment in Medicare and Medicaid, living arrangement (alone, with spouse, with children, with others or in a retirement community), and residential location (metropolitan or non-metropolitan area). Health and clinical characteristics were self-reported and included number of comorbidities (hypertension, myocardial infarction, angina/chronic heart disease, other heart disease, stroke, diabetes mellitus, Parkinson’s disease, emphysema/asthma/chronic obstructive pulmonary disease, rheumatoid arthritis, non-rheumatoid arthritis, osteoporosis/soft bones and cancers other than skin), presence of a developmental, psychiatric or cognitive disorder (mental retardation, Alzheimer’s/dementia or mental/psychiatric disorders), vision impairment, and hearing impairment. In addition, we included an indicator of proxy versus self-response to the survey. We chose not to use specific conditions or comorbidity indices based on claims ICD-9 codes because the assessment periods of these indices partially overlap with indicator-level follow-up periods, instead of preceding follow-up periods.

### Activity limitation stages

Activity limitation stages based on the International Classification of Functioning Disability and Health (ICF) [30] in separate activity of daily living (ADL) and instrumental activity of daily living (IADL) domains were derived from survey data for each respondent. ADL stages include the self-care functions of eating, toileting, dressing, bathing or showering, getting in/out of bed or chairs and walking. IADL stages incorporate the domestic life functions of telephoning, managing money, preparing meals, doing light housework, shopping for personal items and doing heavy housework. Five ADL stages (0–IV) and five IADL stages (0–IV) present a combination of severity and types of disability (Appendix). Stage III was designed as a non-fitting stage to characterize unusual limitation patterns. Methods for ascertaining stage are documented elsewhere [31, 32].

### Statistical analysis

Chi-square tests were used to assess differences in baseline characteristics among the three age groups. Pairwise chi-square tests were applied to statistically significant between-group differences, with the younger and older old compared to the younger old. Receipt of recommended care was expressed as a percent by dividing the number of instances of recommended care received by the number of opportunities. We calculated the weighted percent of receipt of overall (collapsed across the 38 indicators) and indicator-specific recommended care for all age groups combined and for each age group separately. The association between age group and receipt of overall recommended care was assessed first in an unadjusted logistic regression model, and subsequently in multivariable logistic regression. Separate adjusted models were fit for ADL and IADL stages because collinearity precludes including both domains in a single model. Age group and covariates including sex, race and education were assessed at baseline, and other covariates that may vary over time were assessed in the survey cycle immediately preceding indicator the follow-up date. The model applied survey sampling weights and accounted for the complex sampling design and non-independence of multiple eligible indicators for the same individual. Analyses were conducted using SAS 9.4 (SAS Institute, Cary, NC).

## Results

### Sample characteristics

The distribution of the baseline sample (*N* = 30,117) by age group was 16% were younger than age 65 years, 48% aged 65–74 years, and 36% aged 75 years and older. Table 1 lists baseline characteristics by age group. The most striking sociodemographic differences among the age groups were in race/ethnicity, living arrangement and dual enrollment. Compared to the older groups, younger beneficiaries were more likely to be non-Hispanic black (19% vs. 9% and 7%) and Hispanic (11% vs. 8% and 6%), to live with others (24% vs. 5% and 4%), and to be dually-enrolled in Medicaid (44% vs. 11% and 12%).

**Table 1:** Sociodemographic, functional and clinical characteristics of medicare beneficiaries (2001–2008) by age group

Variable	Total *N* = 30,117	Age < 65 N (column weighted %) 5201 (16.3)	Age 65–74 N (column weighted %) 11,289 (47.5)	Age ≥ 75 N (column weighted %) 13,627 (36.2)	*p*-value
Gender					<.0001
Male	13,649 (45.2)	2853 (52.6)	5360 (46.3)	5436 (40.3)	
Female	16,468 (54.8)	2348 (47.4)	5929 (53.7)	8191 (59.7)	
Race/Ethnicity					<.0001
Non-Hispanic White	23,893 (79.2)	3459 (67.3)	9017 (79.9)	11,417 (83.7)	
Non-Hispanic Black	2966 (9.7)	1007 (18.6)	1007 (8.7)	952 (7.0)	
Hispanic	2372 (7.9)	580 (11.2)	913 (8.0)	879 (6.3)	
Other	886 (3.2)	155 (2.9)	352 (3.4)	379 (2.9)	
Living arrangement					<.0001
Retirement community	1905 (5.6)	101 (2.2)	430 (3.6)	1374 (9.7)	
With spouse	14,124 (51.2)	1649 (39.1)	7004 (62.6)	5471 (41.6)	
With children	3158 (9.5)	658 (11.4)	802 (7.0)	1698 (12.1)	
With others	2762 (7.8)	1597 (23.6)	589 (5.2)	576 (4.2)	
Alone	8168 (25.9)	1196 (23.8)	2464 (21.6)	4508 (32.5)	
Dual enrollment in Medicare and Medicaid					<.0001
No	24,292 (83.5)	2409 (56.4)	9984 (89.4)	11,899 (87.8)	
Yes	5825 (16.5)	2792 (43.6)	1305 (10.6)	1728 (12.2)	
Education					<.0001
High school diploma or above	21,252 (72.8)	3527 (69.1)	8543 (77.4)	9182 (68.5)	
No high school diploma	8865 (27.2)	1674 (30.9)	2746 (22.6)	4445 (31.5)	
Living in metropolitan area					<.0001
No	7942 (25.0)	1598 (29.0)	3092 (25.1)	3252 (23.1)	
Yes	22,175 (75.0)	3603 (71.0)	8197 (74.9)	10,375 (76.9)	
Proxy report					<.0001
No	27,492 (92.8)	4277 (87.2)	10,757 (95.4)	12,458 (91.9)	
Yes	2625 (7.2)	924 (12.8)	532 (4.6)	1169 (8.1)	
Vision impairment					<.0001
No	27,674 (92.7)	4621 (87.9)	10,752 (95.8)	12,301 (90.8)	
Yes	2443 (7.3)	580 (12.1)	537 (4.2)	1326 (9.2)	
Hearing impairment					<.0001
No	27,934 (93.5)	4890 (93.8)	10,745 (95.5)	12,299 (90.8)	
Yes	2183 (6.5)	311 (6.2)	544 (4.5)	1328 (9.2)	
Cognitive, developmental and psychiatric disorders^a^					<.0001
No	25,662 (87.3)	2766 (60.9)	10,442 (93.0)	12,454 (91.8)	
Yes	4455 (12.7)	2435 (39.1)	847 (7.0)	1173 (8.2)	
Average number of comorbidities^b^	2.2 ± 0	2.3 ± 0	2.1 ± 0	2.4 ± 0	<.0001
Activity of Daily Living (ADL) Stages					<.0001
0	19,874 (68.3)	2599 (45.4)	8846 (79.9)	8429 (63.5)	
I	5181 (16.5)	1156 (25.2)	1398 (11.8)	2627 (18.9)	
II	2622 (7.9)	656 (13.7)	568 (4.5)	1398 (9.6)	
III	2047 (6.2)	656 (13.5)	417 (3.3)	974 (6.7)	
IV	393 (1.1)	134 (2.3)	60 (0.5)	199 (1.3)	
Instrumental Activity of Daily Living (IADL) stages					<.0001
0	16,911 (59.4)	1339 (23.8)	8169 (74.0)	7403 (56.2)	
I	5332 (17.6)	1063 (24.9)	1670 (14.2)	2599 (18.8)	
II	2979 (9.5)	1046 (22.2)	675 (5.6)	1258 (8.9)	
III	4089 (11.4)	1500 (25.0)	665 (5.3)	1924 (13.2)	
IV	806 (2.1)	253 (4.0)	110 (0.8)	443 (2.9)	

^a^Cognitive, developmental, and psychiatric disorders include: mental retardation, Alzheimer’s/dementia and mental/psychiatric disorder

^b^Number of comorbidities including: hypertension, myocardial infarction, angina/chronic heart disease, other heart disease, stroke, diabetes mellitus, Parkinson’s disease, emphysema/asthma/chronic obstructive pulmonary disease, rheumatoid arthritis, non-rheumatoid arthritis, osteoporosis/soft bones and other (non-skin) cancer

Younger beneficiaries carried a disproportionate burden of developmental, cognitive and psychiatric disorders (39% vs. 7% and 8%). They were significantly less likely to be functionally independent in ADLs (stage 0) compared to the other two older age groups (45% vs. 80% and 64%). Differences in IADL stages were even more striking: only 24% of younger beneficiaries were IADL independent (stage 0) compared to 74% of the younger old and 56% of the older old. They relied more heavily on proxy responses to survey questions and were more likely to be visually impaired.

### Receipt of recommended care by age group across all indicators

In total 20,449 unique beneficiaries were eligible for at least one opportunity for recommended care, including 3756 younger, 7180 younger old and 9513 older old beneficiaries. These beneficiaries triggered 89,076 opportunities for care, with 14,015 for younger beneficiaries, 32,372 opportunities for the younger old, and 42,689 for the older old. As shown in Table 2, eligible younger beneficiaries received recommended care in 64% of the opportunities, in contrast to 73% for the younger old and 75% for the older old.

**Table 2:** Receipt of recommended care among medicare beneficiaries (2001–2008) by age group at the indicator level

	Overall	Age < 65	Age 65–74	Age ≥75
Total number of opportunities for recommended care (unweighted denominator)	89,076	14,015	32,372	42,689
Total number of instances of recommended care received (unweighted numerator)	64,157	8702	23,582	31,873
Weighted percent of recommended care received	72.1%	63.9%	72.7%	74.8%

### Receipt of recommended care by age group by indicator

**Fig. 1:**
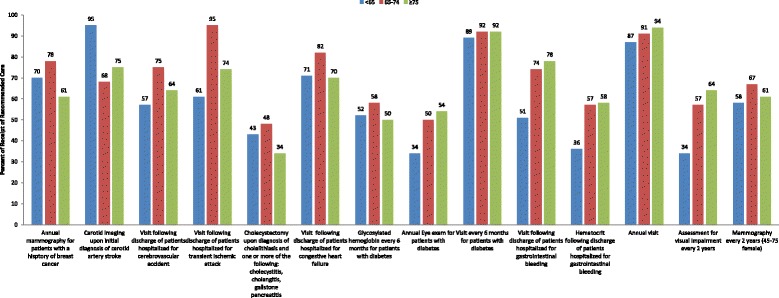
Disparities in receipt of recommended care among younger versus older age groups (<65, 65–74, ≥75)

Table 3 presents the weighted percent of receiving recommended care by age group for each indicator. The Centers for Medicare and Medicaid Services (CMS) prohibits publishing cell size below 11, yielding 30 eligible indicators for comparison, 14 of which had a statistically significant difference (*p* < .05) in receipt of recommended care by age group, shown in Fig. 1. Among these 14 indicators, pair-wise chi-square tests showed younger beneficiaries underused care on 10 indicators when compared to the younger old, and the older old group underutilized care on 5 indicators. Younger beneficiaries outperformed younger old for 1 indicator, while the older old did so for 4 indicators. Notably, younger beneficiaries were less likely than the younger old to have a follow-up visit within 4 weeks following hospital discharge for CVA, TIA and gastrointestinal (GI) bleed, to obtain a hematocrit within 4 weeks following an initial diagnosis of GI bleed, to receive routine care for diabetes (a glycosylated hemoglobin every 6 months, an annual eye exam and a doctor visit every 6 months), and preventive care in general (an annual physician visit, a biennial mammogram and a biennial assessment of visual impairment).

**Table 3:** Receipt of recommended care by indicator among medicare beneficiaries (2001–2008) by age group

Recommended care indicator	Overall *N* = 30,117		Age < 65 5201 (16.3%)	Age 65–74 11,289 (47.5%)	Age ≥ 75 13,627 (36.2%)	*P*-value for difference among age groups
	Raw numerator/denominator	Weighted percent (%)	Weighted percent (%)	Weighted percent (%)	Weighted percent (%)	
Acute Myocardial Infarction (AMI)						
Visit within 4 weeks following discharge of patients hospitalized for acute myocardial infarction	231/298	79	84	79	78	0.748
Cholesterol test every 6 months for patients hospitalized for myocardial infarction who have hypercholesterolemia	224/365	64	71	69	58	0.188
Anemia						
Gastrointestinal workup for patients with iron deficiency anemia no later than 3 months after iron deficiency	355/1112	33	34	37	30	0.273
Hematocrit/hemoglobin between 1 and 6 months following initial diagnosis of anemia	1723/2576	68	67	67	69	0.633
Angina						
Visit within 4 weeks following discharge of patients hospitalized for unstable angina	193/234	83	76	83	86	0.407
Visit every 6 months for patients with chronic stable angina	1826/1940	94	92	93	96	0.135
Follow-up visit or hospitalization within 4 weeks of initial diagnosis of unstable angina	196/236	84	77	82	89	0.150
Lipid profile within 6 months after initial diagnosis of angina	59/767	9	X	14	4	0.0003
Breast Cancer						
Interval from biopsy and definitive therapy less than 3 months for patients with breast cancer and eventual mastectomy	60/79	73	X	70	81	0.273
Mammogram within 3 months preceding an initial diagnosis of breast cancer	110/182	61	X	60	63	0.917
Chest x-ray within 3 months preceding or following initial diagnosis of breast cancer	96/182	51	43	54	51	0.631
Visit within 12 months for breast cancer patients who have undergone mastectomy without cytotoxic chemotherapy	71/71	100	X	100	100	N/A
Mammography every year for patients with a history of breast cancer	416/629	69	70	78	61	0.0004
Cerebrovascular Accident (CVA)						
Carotid imaging within 2 weeks of initial diagnosis for patients hospitalized for carotid artery stroke	235/312	75	95	68	75	<.0001
Interval between carotid imaging and carotid endarterectomy less than 2 months for cerebrovascular accident patients with eventual carotid endarterectomy	112/134	84	X	87	83	0.501
Visit within 4 weeks following discharge of patients for cerebrovascular accident	379/571	67	57	75	64	0.011
Transient Ischemic Attack (TIA)						
Electrocardiogram within 2 days of initial diagnosis of transient ischemic attack	92/621	15	X	16	14	0.748
Interval between carotid imaging and carotid endarterectomy less than 2 months for TIA patients with eventual carotid endarterectomy	45/54	85	X	91	82	0.012
Visit within 4 weeks following discharge of patients hospitalized for transient ischemic attack	184/237	79	61	95	74	<.0001
Visit every year for patients with diagnosis of transient ischemic attack	1540/1596	97	96	97	96	0.740
Cholelithiasis						
Cholecystectomy within 1 month preceding or 3 months following diagnosis of cholelithiasis and one or more of the following: cholecystitis, cholangitis, gallstone pancreatitis	282/699	41	43	48	34	0.030
Chronic Obstructive Pulmonary Disease (COPD)						
Visit every 6 months for patients with chronic obstructive pulmonary disease	4732/5197	91	90	91	92	0.236
Congestive Heart Failure (CHF)						
Chest x-ray within 3 months of initial diagnosis of congestive heart failure	1097/1580	69	72	64	71	0.067
Electrocardiogram within 3 months of initial diagnosis of congestive heart failure	1023/1578	66	67	66	66	0.953
Visit within 4 weeks following discharge of patients hospitalized for congestive heart failure	490/663	74	71	82	70	0.032
Visit every 6 months for patients with congestive heart failure	4142/4527	92	91	93	91	0.201
Depression						
Visit within 2 weeks following discharge of patients hospitalized for depression	95/173	53	49	55	57	0.593
Diabetes Mellitus (DM)						
Glycosylated hemoglobin every 6 months for patients with diabetes	3499/6756	54	52	58	50	<.0001
Eye exam every year for patients with diabetes	3160/6491	49	34	50	54	<.0001
Visit within 4 weeks following discharge of patients hospitalized for diabetes	295/430	68	71	63	70	0.466
Visit every 6 months for patients with diabetes	6185/6756	92	89	92	92	0.036
Gastrointestinal Bleeding						
Visit within 4 weeks following discharge of patients hospitalized for gastrointestinal bleeding	273/373	73	51	74	78	0.001
Hematocrit within 4 weeks following discharge of patients hospitalized for gastrointestinal bleeding	201/373	54	36	57	58	0.025
Follow-up visit within 4 weeks of initial diagnosis of gastrointestinal bleeding	491/676	74	74	77	69	0.195
Hypertension						
Visit within 4 weeks following discharge of patients hospitalized with malignant or otherwise severe hypertension	49/74	63	X	62	76	0.0002
Preventive Care						
Visit every year	17,905/19,535	92	87	91	94	<.0001
Assessment of visual impairment every 2 years	9363/16,759	56	34	57	64	<.0001
Mammography every 2 years for females aged between 45 and 75 (inclusive) years	2728/4240	65	58	67	61	<.0001

Note. According to the Centers for Medicare and Medicaid Services, cell size below 11, marked with an X, is not permitted for publication

Compared to the younger old, the older old beneficiaries were less likely to receive follow-up care for CHF, TIA and CVA after hospital discharge. However, the older old were more likely to attend an annual doctor visit, to have a biennial eye exam, and to receive eye exam for diabetes.

### Factors associated with receipt of recommended care

Table 4 displays the association between age group and receipt of recommended care in a bivariate logistic regression model and multivariable logistic regression models that included ADL and IADL stages separately. In the unadjusted model, the odds of receiving overall recommended care was 34% lower among younger beneficiaries, but 11% higher among older old beneficiaries, each compared to the younger old.

**Table 4:** Logistic Regression Models Predicting Receipt of Recommended Care among Medicare Beneficiaries (2001–2008)

Variables	Model 1		Model 2 with ADL stages		Model 2 with IADL stages	
	OR (95% CI)	*p*-value	OR (95% CI)	*p*-value	OR (95% CI)	*p*-value
Age (ref: 65–74)		<.0001		<.0001		<.0001
<65	0.66 (0.62–0.71)	<.0001	0.75 (0.70–0.80)	<.0001	0.75 (0.69–0.80)	<.0001
≥75	1.11 (1.07–1.16)	<.0001	1.15 (1.10–1.20)	<.0001	1.15 (1.10–1.20)	<.0001
Gender (ref: female)						
Male			0.86 (0.82–0.90)	<.0001	0.86 (0.82–0.90)	<.0001
Race/Ethnicity (ref: Non-Hispanic White)				0.005		0.005
Hispanic			0.95 (0.87–1.05)	0.337	0.95 (0.86–1.04)	0.277
Non-Hispanic Black			0.88 (0.82–0.95)	0.0004	0.88 (0.82–0.95)	0.0005
Other			0.96 (0.86–1.08)	0.527	0.96 (0.85–1.08)	0.464
Education (ref: high school diploma)						
No high school diploma			0.85 (0.81–0.89)	<.0001	0.85 (0.81–0.89)	<.0001
Living Arrangement (ref: live with spouse)				<.0001		<.0001
Alone			0.88 (0.83–0.92)	<.0001	0.87 (0.83–0.92)	<.0001
Retirement community			0.95 (0.87–1.03)	0.199	0.95 (0.87–1.03)	0.188
With children			0.77 (0.72–0.83)	<.0001	0.77 (0.72–0.83)	<.0001
With others			0.82 (0.75–0.89)	<.0001	0.83 (0.76–0.90)	<.0001
Residential Location (ref: Non-Metropolitan location)						
Metropolitan location			1.14 (1.09–1.18)	<.0001	1.13 (1.08–1.18)	<.0001
Dual Enrollment in Medicare and Medicaid (ref: Medicare only)						
Dual enrollment			1.06 (1.00–1.13)	0.056	1.06 (1.00–1.13)	0.072
Proxy Response (ref: no)						
Proxy			0.87 (0.81–0.93)	<.0001	0.90 (0.84–0.96)	0.003
Conditions (ref: no)						
Vision impairment			1.01 (0.94–1.09)	0.731	1.02 (0.94–1.10)	0.694
Hearing impairment			0.95 (0.88–1.02)	0.162	0.96 (0.89–1.03)	0.242
Cognitive, developmental, and psychiatric disorders*			0.89 (0.83–0.94)	<.0001	0.90 (0.84–0.96)	<.0001
Sum of comorbidities**			1.12 (1.11–1.14)	<.0001	1.12 (1.10–1.13)	0.001
Stage (ref: Stage 0)				<.0001		<.0001
Stage I			0.92 (0.88–0.97)	0.003	0.99 (0.94–1.05)	0.763
Stage II			0.87 (0.81–0.93)	<.0001	0.89 (0.84–0.96)	0.001
Stage III			0.80 (0.74–0.87)	<.0001	0.87 (0.81–0.93)	<.0001
Stage IV			0.64 (0.54–0.76)	<.0001	0.69 (0.61–0.78)	<.0001

*Note*: Ref=reference category. For a variable that has more than two categories, a total *p* value of the variable is reported

Model 1 is adjusted only for age group; model 2’s are further adjusted for sociodemographics, health and clinical characteristics and ADL stages and IADL stages separately

* Cognitive, developmental, and psychiatric disorders include: mental retardation, Alzheimer's/dementia, and mental/psychiatric disorder

** Sum of comorbidities include: hypertension, myocardial infarction, angina/chronic heart disease, other heart disease, stroke, diabetes mellitus, Parkinson's disease, emphysema/asthma/chronic obstructive pulmonary disease, rheumatoid arthritis, non-rheumatoid arthritis, osteoporosis/soft bones, and other (non-skin) cancer

Model estimates for separate stage systems were similar (Table 4), after excluding less than 2% of missing cases. In the multivariable model adjusted for ADL stages, the association (OR) between younger age and receipt of recommended care was attenuated to 0.75. Male gender, black race, less than high school education, living alone, with children or with others (each compared to living with spouse), proxy response and having developmental, cognitive or psychiatric disorders were all independently associated with underuse of recommended care. Living in a metropolitan area and a greater number of comorbidities were associated with appropriate care. Both ADL and IADL stages showed ordered associations with receipt of recommended care. Compared to no ADL limitations (stage 0), the likelihood of receiving recommended care declined with higher ADL stages, with ORs (95% CIs) across stages I–IV at 0.92 (0.88–0.97), 0.87 (0.81–0.93), 0.80 (0.74–0.87) and 0.64 (0.54–0.76), respectively. A similar pattern held for IADL stages.

## Discussion

Research on the appropriate use of health services by younger Medicare beneficiaries remains quite limited [3]. In this nationally representative study of community dwelling Medicare beneficiaries, we found that those younger than 65 compared to those 65–74 years of age had a higher proportion of characteristics conventionally associated with social disadvantage. Such characteristics include being non-Hispanic Black, living with disabilities, lower educational achievement and non-metropolitan residency. Even after adjusting for these factors and further adjusting for dual enrollment in Medicare and Medicaid, cognitive, developmental or psychiatric disorders and vision impairment, we found substantially reduced use of recommended care by younger Medicare beneficiaries. In contrast, the older old group was slightly more likely than the younger old to receive recommended care.

Our results are consistent with previous reports on younger beneficiaries with respect to the proportion of those who were non-Hispanic black, who were eligible for Medicare and Medicaid [5, 33] and who self-reported to have cognitive, developmental or psychiatric disorders [6]. Younger beneficiaries demonstrated a higher prevalence of self- or proxy- reported dependencies in ADLs and IADLs in our study than previously reported [34]. The results suggest that activity limitations of younger Medicare beneficiaries have not improved over time, supporting need for interventions.

Although it has been reported that younger Medicare beneficiaries significantly underuse preventive care compared to older beneficiaries [4], our study was able to quantify the extent of such deficits. We found the most striking deficiencies across the three prevention indices, routine care for diabetes and post-discharge follow-up for CVA and TIA. Inadequate care, particularly for chronic conditions, suggests that the current service delivery model that centers on acute illness [35] does not meet current needs for prevention and chronic conditions. The reorientation of Medicare to the management of chronic illness and the amelioration of activity limitation could improve the care and reduce costs for chronically ill beneficiaries [36]. Appropriate use of preventive services, medication management and behavioral interventions have been proposed as promising strategies for reducing severity of chronic conditions and their complications [3].

Younger beneficiary status was an independent predictor of underuse in the adjusted model, possibly due to the operation of unknown factors influencing underuse in this population, such as infrequent contact with the health system, especially outpatient services. A post-hoc analysis revealed that among beneficiaries with a cognitive, developmental or psychiatric disorder, the three age groups made similar numbers of outpatient visits (*Median* = 2.2, 2.1 and 1.9 respectively); in contrast, among beneficiaries without those disorders, younger beneficiaries visited a doctor more often than the younger old and older old beneficiaries (*Median* = 2.1 vs. 1.0 and 1.5). The excess office visits made by younger beneficiaries were likely due to Medicare eligible conditions other than cognitive, developmental or psychiatric disorders. These findings suggest that a greater number of office visits does not necessarily translate into adequate care for younger beneficiaries. One explanation for the paradox is that specialists may not make recommendations for preventive care outside their area of specialty. We speculate that improved care coordination among mental health, primary care and specialty care providers may contribute to a better understanding of patients’ comprehensive care needs and making critical recommendations.

In contrast, older old beneficiaries had a slightly better chance to get recommended care than the younger old, all else equal. This is consistent with our post-hoc finding that on average older old beneficiaries visited their doctors more often than the younger old. We found greater comorbidity associated with greater likelihood of receiving appropriate care, similar to published reports [26, 37]. Increased use of recommended care for both groups is likely due to their frequent office visits leading to a greater chance to fulfill care requirements.

As expected, non-Hispanic black race, less than high-school education, non-Metropolitan residence and disability independently predicted underuse of care. Although reversed racial disparity has been reported [27], likely due to selection bias of the samples [38], different sets of quality of care indicators studied, and use of claims versus self-reported data, underuse of medical care among racial minorities is more accentuated in literature [21, 39]. Improving surveillance data systems, creating a culturally-competent medical workforce and recruiting minority health professionals have emerged as strategies to address racial/ethnic differences in health and health care [40, 41]. Lower socioeconomic position [42] and rural settings [43, 44] diminish the chance to obtaining cancer prevention services. Removal of access barriers to care, especially financial barriers, was endorsed as central to create equity in health outcomes across different socioeconomic groups [45]. Availability of services, knowledge or physician recommendations of needed care and transportation are often reported factors underlying the geographic disparities in care and are points to address in interventions [43, 44] Greater use of home care in rural areas was also reported [46]. Future research may investigate population-level utilization of a wide range of health services. Disability is a known risk factor for underuse of certain care among Medicare beneficiaries excluding younger beneficiaries [24, 25]. This is also reflected in our study, which found a monotonic increase in care disparities with higher activity limitation stages (greater severity). Physical barriers, lack of professional assistance and social support, as well as experiences of distress likely influence service underuse [47, 48]. Resource reallocation targeting disabled individuals may aid their access to care and increase use of recommended care. Furthermore, since functional decline after hospitalization is fairly common [49], establishing care continuity in communities after hospital discharge can be critical for disabled persons.

We studied three Medicare age groups who likely occupy different positions in a social hierarchy and differ in their health status and utilization of health services. Such comparison is useful in identifying a disadvantaged population and its care needs, which subsequently informs resource reallocation to achieve greater equity. The study has several limitations. This study does not answer the question why younger beneficiaries underuse recommended care. The mechanism can be explained by access barriers to care, care not recommended by providers, or care recommended by providers but was not sought by the patient. For instance, some beneficiaries did not seek or comply with recommended care because of their limited health literacy or knowledge about their care plans [50, 51]. It has also been reported that providers tend to downplay the importance of healthy behaviors and disease prevention in the lives of their disabled patients [47]. Due to data limitation, we were not able to incorporate these potential causes for failure of care compliance in our analysis. We recommend in-depth observational studies that explore patient-doctor encounters to determine the causes of underuse and what types of appropriate preventions should be in place. Asch’s indicator system reflects care needs of highly prevalent conditions among the elderly population. These indicators may not reflect all care needs of younger beneficiaries, especially those experiencing cognitive, developmental or psychiatric disorders. Indicators that address the care for prevalent diseases in younger beneficiaries are highly desirable. Stratified analysis of receipt of recommended care among beneficiaries with versus without psychiatric disorders may also be considered, since persons who have been admitted for mental disorders tend to have poorer quality of care and higher mortality in somatic diseases, compared to persons who only have somatic diseases [52]. We acknowledge the likelihood of residual confounding in socioeconomic, comorbidity and to a lesser extent disability, measures. It is possible that even after controlling for all these variables, the reason of underuse among younger beneficiaries is that they are still sicker and more disadvantaged, rather than an independent effect of younger beneficiary status. Although there may be geographic variations in receipt of recommended care, MCBS is not powered to investigate state-level estimates. MCBS claims data (2002–2010) used in this study are not the most recent; however, the structure of the Medicare program eligibility for those under 65 has not changed, and the historical data matches the period when Asch’s indicators were developed. Due to incomplete claims data from beneficiaries enrolled in a managed care program, our results only apply to the fee-for-service Medicare population. Even though we combined eight beneficiary cohorts to compensate for small sample sizes associated with certain indicators, some indicators could not be addressed in the younger beneficiaries since cell sizes were still too small to report.

## Conclusions

Our study has identified social and medical vulnerabilities of younger Medicare beneficiaries, and their lack of overall and specific type of care. Our results based on improved indicator metrics corroborated previous findings of potential influences on health service underutilization. CMS (Quality Strategy 2016) envisions care as valued-based: person-centered, cost-efficient and health-promoting [53]. It sets effective communication and coordination of care, prevention and treatment of chronic diseases, and partnership with communities to promote healthy living as among its goals, and eliminating racial and ethnic disparities and strengthening infrastructure and data systems as part of its foundational principles. Our findings provide evidence for the need of interventions that may bridge the health equity gap in the Medicare population.
